# Relationship between PI3K/mTOR/RhoA pathway-regulated cytoskeletal rearrangements and phagocytic capacity of macrophages

**DOI:** 10.1590/1414-431X20209207

**Published:** 2020-06-03

**Authors:** H.R. Bao, J.L. Chen, F. Li, X.L. Zeng, X.J. Liu

**Affiliations:** Department of Gerontal Respiratory Medicine, The First Hospital of Lanzhou University, Lanzhou, Gansu, China

**Keywords:** Phagocytosis, Cytoskeleton, PI3K, mTOR, RNA interference

## Abstract

The objective of this study was to investigate the relationship between PI3K/mTOR/RhoA signaling regulated cytoskeletal rearrangements and phagocytic capacity of macrophages. RAW264.7 macrophages were divided into four groups; blank control, negative control, PI3K-RNAi, and mTOR-RNAi. The cytoskeletal changes in the macrophages were observed. Furthermore, the phagocytic capacity of macrophages against *Escherichia coli* is reported as mean fluorescence intensity (MFI) and percent phagocytosis. Transfection yielded 82.1 and 81.5% gene-silencing efficiencies against PI3K and mTOR, respectively. The PI3K-RNAi group had lower mRNA and protein expression levels of PI3K, mTOR, and RhoA than the blank and negative control groups (Р<0.01). The mTOR-RNAi group had lower mRNA and protein levels of mTOR and RhoA than the blank and the negative control groups (Р<0.01). Macrophages in the PI3K-RNAi group exhibited stiff and inflexible morphology with short, disorganized filopodia and reduced number of stress fibers. Macrophages in the mTOR-RNAi group displayed pronounced cellular deformations with long, dense filopodia and an increased number of stress fibers. The PI3K-RNAi group exhibited lower MFI and percent phagocytosis than blank and negative control groups, whereas the mTOR-RNAi group displayed higher MFI and percent phagocytosis than the blank and negative controls (Р<0.01). Before and after transfection, the mRNA and protein levels of PI3K were both positively correlated with mTOR and RhoA (Р<0.05), but the mRNA and protein levels of mTOR were negatively correlated with those of RhoA (Р<0.05). Changes in the phagocytic capacity of macrophages were associated with cytoskeletal rearrangements and were regulated by the PI3K/mTOR/RhoA signaling pathway.

## Introduction

Chronic obstructive pulmonary disease (COPD) poses a serious threat to human health and is showing a gradual rise in incidence and mortality rates (1). It is expected to rank third in the world in terms of incidence and mortality rates by 2020, imposing huge economic burdens (2). COPD is a complex and heterogeneous disease at the clinical and cellular levels, with airflow obstruction and a progressive decline in lung function, including small-airway obstructions and emphysema (3). Recent studies found that a decline in the phagocytic capacity of alveolar macrophages (AM) reduced their bacterial eradication rate and thus, led to frequent acute exacerbations in patients with COPD and asthma (4,5). AM are capable of immune surveillance and pathogen eradication and serve as the first line of defense in the human immune system (6). Therefore, it is important to investigate the exact mechanism underlying the weakened phagocytic capacity of AM, in order to understand the acute exacerbations of COPD.

It is well known that the process of phagocytosis is accompanied by dramatic changes in actin cytoskeletal rearrangements and phosphoinositide 3-kinases (PI3K)-related signaling pathways (7,8). It is also well known that PI3Ks are a family of lipid kinase proteins that regulate the growth, proliferation, and cytoskeletal rearrangements of cells (9). Mammalian target of rapamycin (mTOR) is a serine/threonine protein kinase present downstream to PI3K that regulates cell growth, migration, metabolism, and cytoskeletal rearrangements (10,11). Ras homolog gene family member A (Rho A) is a small-molecular weight protein present downstream to mTOR. It affects the phagocytic functions of cells by regulating the assembly of actin and myosin filaments as well as cell adhesion, but it needs to be phosphorylated into p-RhoA to exercise its functions (12). Freeman et al. (13) found that PI3K-mTOR signaling pathway regulates the phagocytic capacity of macrophages, while mTOR modulates cytoskeletal rearrangements by regulating its downstream protein RhoA so that it can be activated via phosphorylation into p-RhoA (14 [Bibr B15]). However, the relationship of PI3K/mTOR/RhoA signaling pathway with cytoskeletal rearrangements and phagocytic capacity of macrophages is rarely reported. Therefore, we inhibited the PI3K/mTOR/RhoA signaling pathway via RNA interference (RNAi) to elucidate its relationship with cytoskeletal rearrangements and phagocytic capacity of macrophages.

## Material and Methods

### Cell culture and experimental groups

RAW264.7 cells (The Cell Bank of Type Culture Collection of Chinese Academy of Sciences, China) were sub-cultured in Dulbecco's modified Eagle medium (DMEM, Hyclone Laboratories, Inc., USA) containing 10% fetal bovine serum (FBS, Clark Bioscience, USA) in 5 % CO_2_ at 37°C, and were divided into four experimental groups as follows: blank control, negative control, PI3K-RNAi, and mTOR-RNAi. The cells in the negative control group were transfected with shRNA (random sequence), while the cells in PI3K-RNAi and mTOR-RNAi groups were transfected with PI3K-shRNA and mTOR-shRNA (Guangzhou Saiye Biotechnology Co., Ltd., China), respectively. The cells in the blank group were not transfected with any vector. All experiments were repeated three times.

### Lipofection of RAW264.7 cells

Plasmids were isolated in strict accordance with the endotoxin-free plasmid extraction kit (Tiangen Biotech Co., Ltd., China). RAW264.7 cells grown to logarithmic phase were inoculated at 2×10^5^ cells/well into a 6-well plate containing OPTI-MEM medium (Gibco, Invitrogen Corp., USA) and transfected with Lipofectamine^TM^ 2000 (Invitrogen Corp., USA) /plasmid DNA complex at a ratio of 2.5:1. After being incubation in 5% CO_2_ at 37°C for 6 h, the OPTI-MEM medium (Gibco, Invitrogen Corp.) was replaced with DMEM complete medium and the cells were further incubated for 72 h. Transfection efficiency was determined by western blot analysis examining the shRNA with the highest transfection efficiency for PI3K and mTOR genes, respectively.

### Construction of lentiviral vectors

PI3K-shRNA, mTOR-shRNA, and negative-control shRNA (Guangzhou Saiye Biotechnology Co., Ltd.), which had the highest transfection efficiencies, were subjected to lentiviral packaging and titration (Guangzhou Saiye Biotechnology Co., Ltd.). All vectors had titers greater than 1×10^8^ TU/mL. Cells grown to logarithmic phase were inoculated into a 6-well plate at 1×10^5^ cells/well. Each of the wells was supplemented with 6 μg/mL of polybrene (Guangzhou Saiye Biotechnology Co., Ltd.), and the cells were transfected at a multiplicity of infection (MOI: the lentivirus-to-cell ratio) of 100. After being transfected for 24 h in 5% CO_2_ at 37°C, the virus-containing medium was replaced with DMEM complete medium and the cells were further incubated for 72 h before observing the expression of enhanced green fluorescent protein (EGFP) under an inverted fluorescence microscope (CKX41, Olympus Co., Japan). Furthermore, the cells were harvested and divided into two portions: one portion was used for the determination of gene-silencing efficiency against PI3K and mTOR, while the other portion was sub-cultured.

### Determination of mRNA levels by real-time quantitative PCR

The total RNA samples extracted from cells of the different groups were subjected to cDNA synthesis using the PrimeScript™ RT reagent Kit (TaKaRa Bio., Inc., Japan), followed by the determination of mRNA levels of PI3K, mTOR, and RhoA according to manufacturer's instructions. Their primers were as follows: PI3K: upstream primer: 5′CCCATGGGACAACATTCCAA3′, downstream primer: 5′CATGGCGACAAGCTCGGTA3′; mTOR: upstream primer: 5′CCCGGACAAGGACAGACTCCTA3′, downstream primer: 5′GGTTTCACCAAACCGTCTCCA3; RhoA: upstream primer: 5′CAGCAAGGACCAGTTCCCAGA3′, downstream primer: 5′AGCTGTGTCCCATAAAGCCAACTC3′. PCR amplification was carried out under the following conditions: pre-denaturation at 95°C for 30 s, followed by 40 cycles of denaturation at 95°C for 5 s and annealing and extension at 60°C for 34 s. The relative expression level of each target gene was calculated using the 2^-△△Ct^ method.

### Western blot assay of protein expressions

The cell suspension was centrifuged at 2000 *g* for 5 min at 4°C, and the supernatant was discarded. Ice-cold PBS was added to the cell pellet, and washed and centrifuged the cells at 2000 *g* for 5-7 min at 4°C, and the supernatant was discarded, and repeated three times. Ice-cold lysis buffer (Beijing Solarbio Science & Technology Co., Ltd., China) was added to the cell pellet. The contents were agitated in microfuge tubes for 30 min at 4°C. The tubes were centrifuged at 16,000 *g* for 20 min at 4°C. The supernatant was collected in fresh tubes and placed on ice, and was used for the measurement of protein concentrations via bicinchoninic acid (BCA) assay. The protein samples were then denatured in a water bath for 5 min and 20 μL each were loaded for separation by sodium dodecyl sulfate-polyacrylamide gel electrophoresis (SDS-PAGE, Beijing Solarbio Science & Technology Co., Ltd.). The separated proteins were transferred by wet transfer method onto a PVDF membrane, which was then blocked with 5% skim milk (Beijing Solarbio Science & Technology Co., Ltd.) for 30 min. The membrane was subsequently incubated overnight at 4°C (in the refrigerator) with the following primary antibodies: rabbit anti-PI3K p85α antibody (Abcam plc., England) (1:1500), rabbit anti-mTOR antibody (ImmunoWay Biotechnology Co., USA) (1:500), mouse anti-RhoA antibody and p-RhoA antibody (Abcam plc.) (1:500), and mouse anti-β-actin antibody (GeneTex, Inc., USA) (1:1500) (1:2500). The membrane was then incubated at room temperature for one hour with goat anti-rabbit IgG (ImmunoWay Biotechnology Co.) (1:5000) and goat anti-mouse IgG (ImmunoWay Biotechnology Co.) (1:5000). The membrane was then exposed to the film after being incubated with enhanced chemiluminescence (ECL) substrate (Millipore, Merck KGaA, Germany). ImageJ (https://imagej.net) was used for quantification of gray intensity. The gray value of each protein band was determined by calculating the relative expression levels of each target protein, which was defined as the gray-value ratio of the target protein to the internal reference protein.

### Observation of cytoskeletons

The cells in different groups were inoculated onto coverslips in a 24-well plate and incubated in the dark at 37°C for 6 h with 300 μL/well of fluorescein isothiocyanate (FITC)-labeled *E. coli* (final concentration: 4 mg/mL) (Invitrogen, Corp.). Each well was then supplemented with 100 μL of 4% trypan blue (Invitrogen Corp.) to quench the extracellular fluorescence of FITC-labeled *E. coli* for one minute. After washing with phosphate-buffered saline (PBS, Beijing Solarbio Science & Technology Co., Ltd.) thrice, the supernatant was discarded, while the remaining cells were fixed with 4% paraformaldehyde (Beijing Solarbio Science & Technology Co., Ltd.) for 30 min and washed 3 times with PBS prior to being stained with 200 μL/well of rhodamine-labeled phalloidin (Cytoskeleton, Inc. USA) at room temperature for one hour. The cells were then washed thrice with PBS and mounted for subsequent observation and imaging under the confocal laser scanning microscope (Carl Zeiss AG, Germany).

### Determination of the phagocytic capacity against FITC-labeled *E. coli*

The plates containing 1×10^5^ cells/well were incubated in the dark for 6 h with FITC-labeled *E. coli* suspension (final concentration: 0.04 mg/mL). Each well was then supplemented with 4% trypan blue to quench the extracellular fluorescence of FITC-labeled *E. coli*. The cells were then harvested via centrifugation (16,000 *g* for 20 min at 4^o^C) for determining the mean fluorescence intensity (MFI) and percentage of phagocytic cells positive for FITC-labeled *E. coli* (percent phagocytosis) using the Mx3000p Flow Cytometer (Becton Dickinson Co., USA). Higher MFI and percent phagocytosis indicate greater phagocytic capacities.

### Statistical analysis

All data were analyzed using the software SPSS ver. 22.0 (IBM, USA). The measurement data are reported as means±SD. The pairwise comparison between groups was carried out using one-way analysis of variance (ANOVA) and LSD *t*-test. Correlation was tested via Pearson's linear correlation analysis. P<0.05 indicated a statistically significant difference.

## Results

### Transfection and gene-silencing efficiencies of PI3K-RNAi and mTOR-RNAi

**Figure 1:**
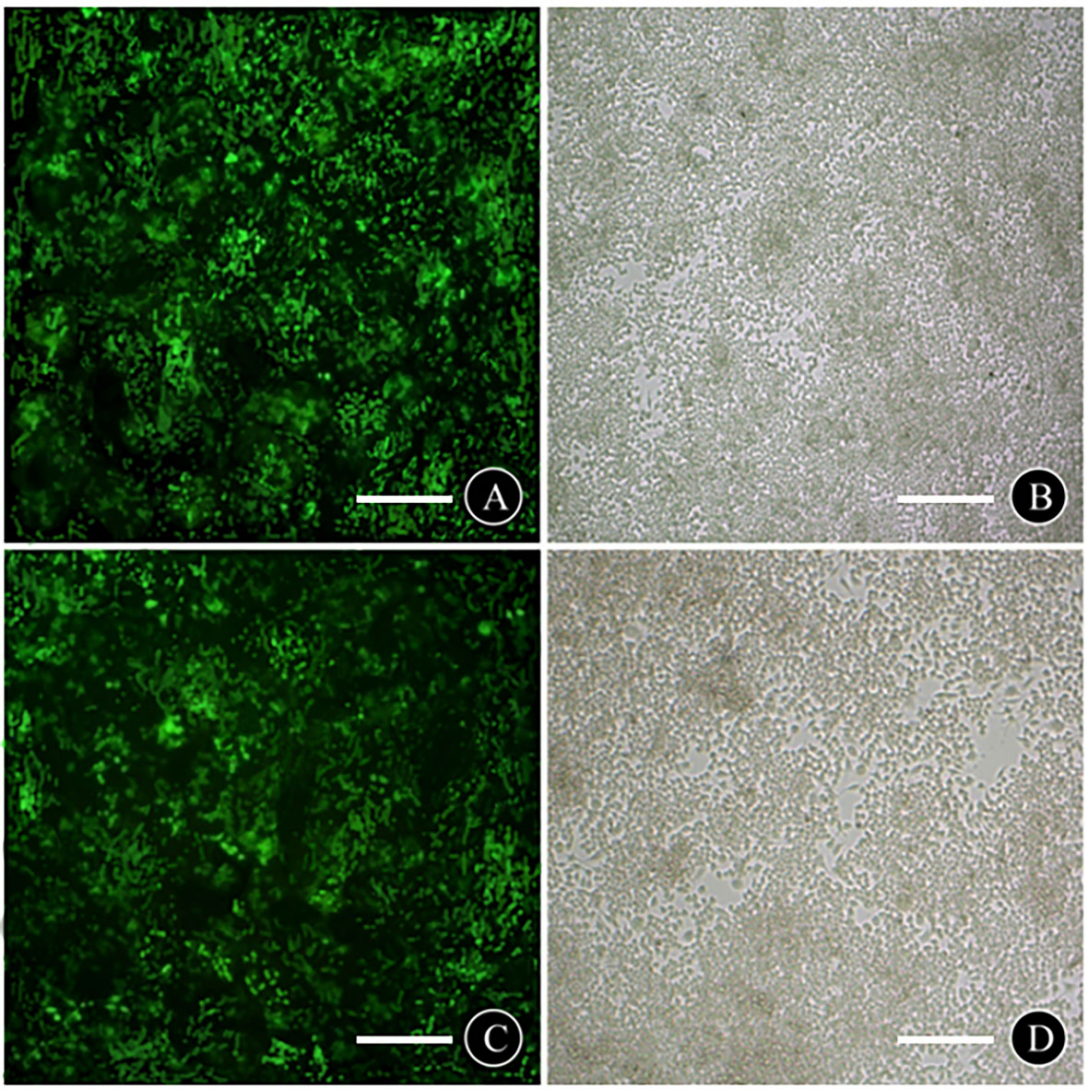
Transfection efficiency of cells in PI3K-RNAi and mTOR-RNAi groups after 72 h transfection. **A**, PI3K-RNAi fluorography; **B**, PI3K-RNAi white light graph; **C**, mTOR-RNAi fluorography; **D**, mTOR-RNAi white light graph. Scale bar, 100 μm.

After 72 h of transfection, EGFP could be observed on transfected cells under the inverted fluorescence microscope with transfection efficiencies >80% (Figure 1) and gene-silencing efficiencies of 82.1±2.1 and 81.5±2.3% for PI3K-RNAi and mTOR-RNAi, respectively. A significant decline in the protein levels of PI3K and mTOR indicated successful silencing of the target genes.

### mRNA and protein expressions of PI3K, mTOR, and RhoA, as well as protein expression of p-RhoA

**Figure 2:**
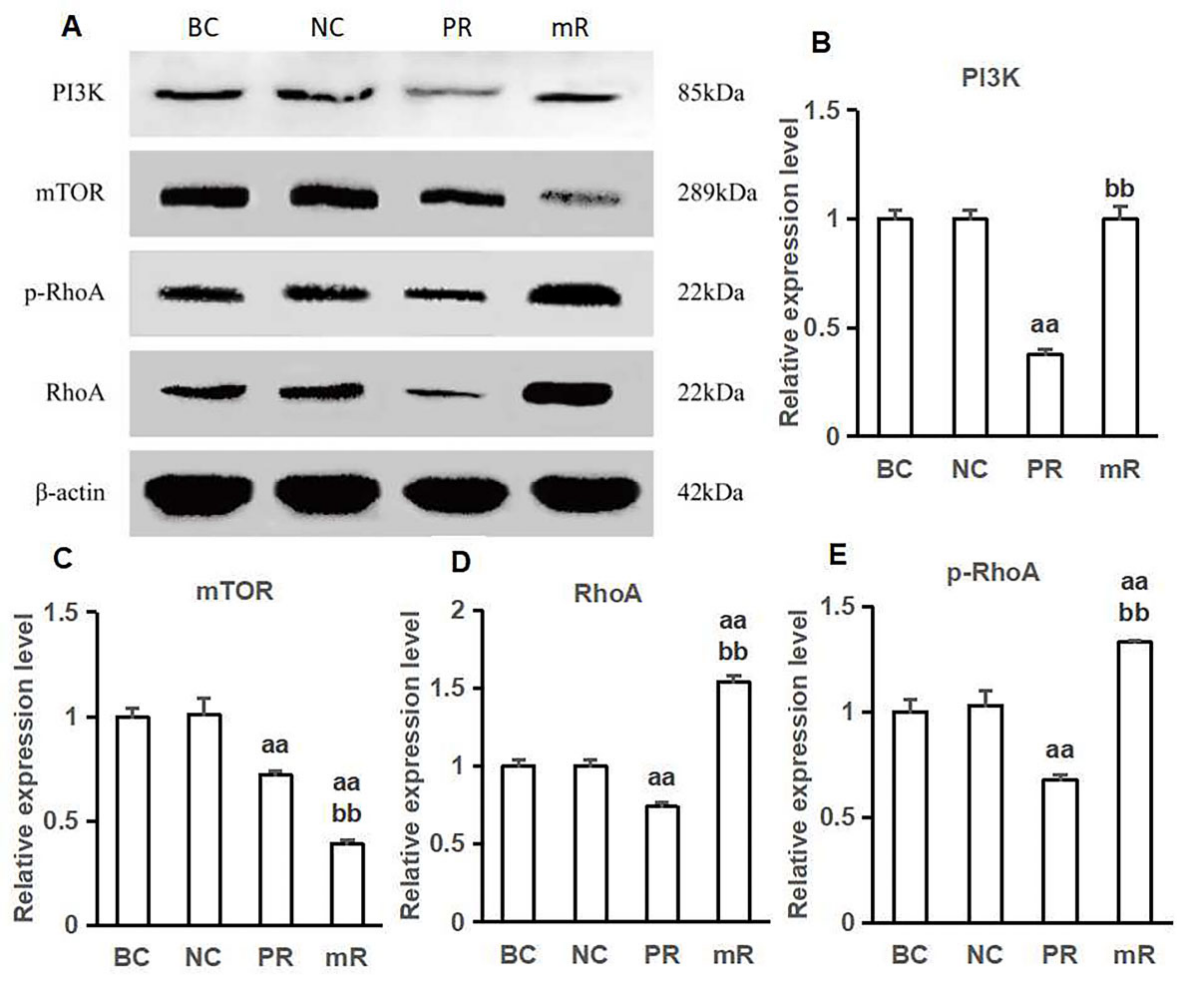
Expression of PI3K, mTOR, RhoA, and p-RhoA proteins in each group. **A**, Western blot test for the expression of PI3K, mTOR, RhoA, and p-RhoA proteins. **B**–**E**, Expression levels of PI3K, mTOR, RhoA, and p-RhoA proteins in each group respectively. ^aa^P<0.01, compared with the blank control group (BC) and the negative control group (NC); ^bb^P<0.01, compared with the PI3K-RNAi group (PR) (ANOVA and LSD *t*-test). mR: mTOR-RNAi group.

The mRNA and protein expression levels of PI3K, mTOR, and RhoA, as well as the phosphorylation levels of p-RhoA protein in the cells from the PI3K-RNAi group were lower than those in the cells from the blank and negative control groups (Р<0.01). Both mRNA and protein expression levels of mTOR in the cells from the mTOR-RNAi group were lower than those in the cells from the blank control and negative control groups (P<0.01), while the mRNA and protein expression levels of RhoA, as well as the phosphorylation level of p-RhoA protein were higher than those in the blank and negative control groups (Р<0.01) (Table 1 and Figure 2).

**Table 1:** mRNA relative expression levels of PI3K, mTOR, and RhoA in each group (mean±SD).

Group	PI3K	mTOR	RhoA
Blank control	1.00±0.0	1.00±0.00	1.00±0.00
Negative control	0.98±0.05	1.01±0.11	0.99±0.13
PI3K-RNAi	0.29±0.07^aa^	0.54±0.13^aa^	0.59±0.06^aa^
mTOR-RNAi	1.02±0.07^bb^	0.30±0.08^aabb^	1.40±0.21^aabb^

^aa^P<0.01 compared with the blank and negative control groups; ^bb^P<0.01 compared with the PI3K-RNAi group (ANOVA and LSD *t*-test).

### Cytoskeletal changes in different groups

**Figure 3:**
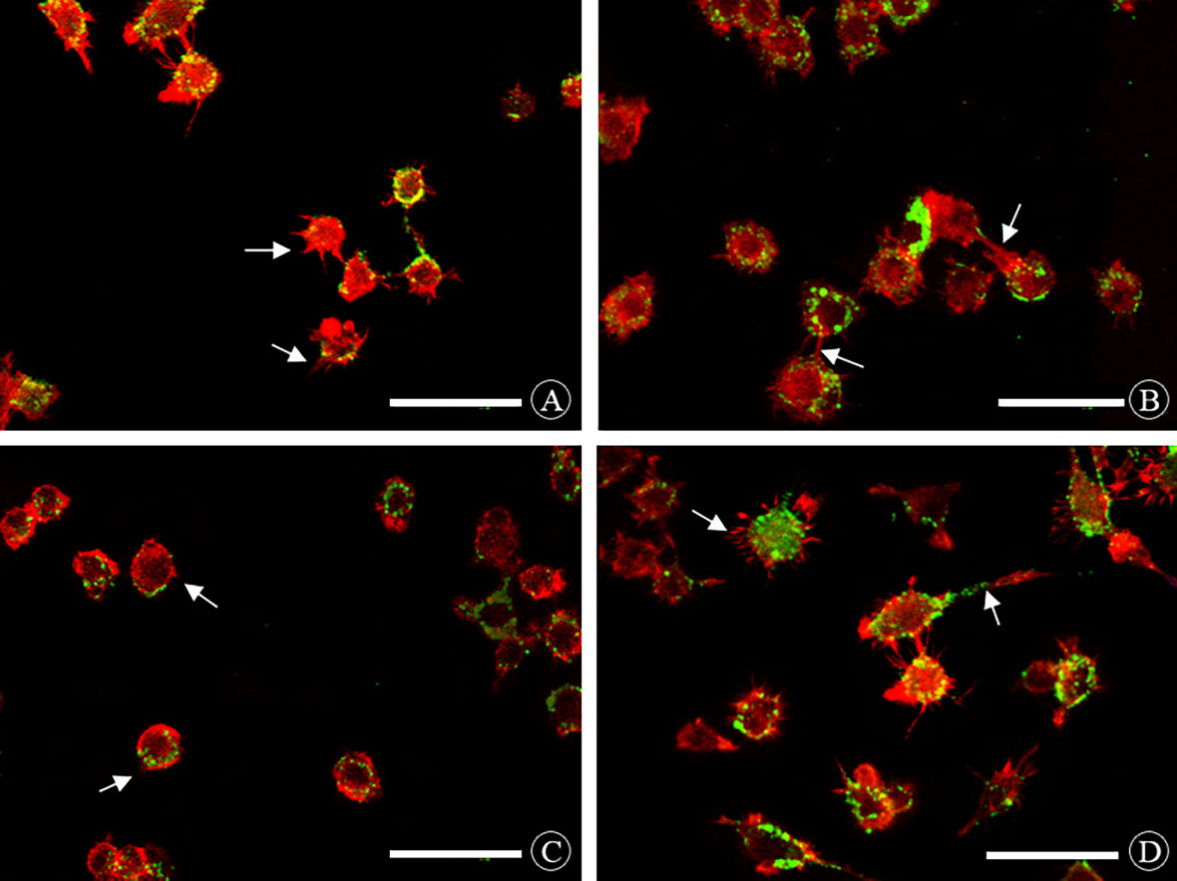
Cytoskeleton morphology of each group under laser confocal microscope (×400, scale bar, 15 μm). **A**, Blank group: protruding long filamentous pseudopodium, cell deformation was obvious. **B**, Negative control group: protruding long filamentous pseudopod, cell deformation was obvious. **C**, PI3K-RNAi group: short protruding pseudopod, cell deformation was poor. **D**, mTOR-RNAi group: longer and denser protruding pseudopod, cell deformation was more obvious. Red fluorescence: rhodamine labeled phalloidin; Green fluorescence: FITC-*E.coli*. White arrow: pseudopodia.

The cells from the PI3K-RNAi group had a stiff and inflexible morphology with short, disorganized filopodia and a relatively smaller number of stress fibers than the cells in the blank group. The cells from mTOR-RNAi group displayed even more pronounced cellular deformation with long, slender, and dense filopodia, as well as a significantly greater number of stress fibers than the cells in the blank control group. Cells from both the blank and negative control groups showed relatively prominent cellular deformations with longer filopodia and a greater number of stress fibers (Figure 3).

### Phagocytic capacity against FITC-labeled *E. coli*

The cells from PI3K-RNAi group showed lower MFI and lower phagocytosis than the cells from the blank and negative control groups (P<0.01), whereas the cells from mTOR-RNAi group had significantly higher MFI and higher phagocytosis than the cells from the blank and negative control groups (P<0.01) (Table 2).

**Table 2:** Phagocytic capacity against FITC-labeled *E. coli* in each group (mean±SD).

Group	MFl	Phagocytosis (%)
Blank control	11733.0±935.0	79.97±1.07
Negative control	10983.0±954.0	79.60±1.17
PI3K-RNAi	7435.0±705.0^aabb^	70.73±2.66^aabb^
mTOR-RNAi	18583.0±1090.0^aabb^	87.72±1.58^aabb^

^aa^P<0.01 compared with the blank group, ^bb^P<0.01 compared with the negative control (ANOVA and LSD *t*-test). MFI: mean fluorescence intensity.

### Correlation analysis

Before and after transfection, the mRNA and protein expression levels of PI3K were positively correlated with that of mTOR (Р<0.05); the mRNA and protein expression levels of PI3K were positively correlated with that of RhoA and p-RhoA (Р<0.05); the mRNA and protein expression levels of mTOR were negatively correlated with that of RhoA and p-RhoA (Р<0.05) (Table 3). The mRNA and protein expression levels of PI3K and RhoA, as well as the expression level of p-RhoA protein were positively correlated with MFI and percent phagocytosis (Р<0.05); whereas mRNA and protein expression levels of mTOR were negatively correlated with MFI and percent phagocytosis (Р<0.05) (Table 4).

**Table 3:** Analysis of the correlation between PI3K, mTOR, and RhoA mRNA and proteins in each group (r value).

Group	RhoA		p-RhoA protein	mTOR	
	mRNA	Protein		mRNA	Protein
**Blank control**					
PI3K					
mRNA	0.88*	0.93**	0.96**	0.94**	0.98**
protein	0.83*	0.97*	0.97**	0.83*	0.93**
mTOR					
mRNA	-0.86*	-0.96**	-0.94**	-	-
protein	-0.94**	-0.97**	-0.99**	-	-
**Negative control**					
PI3K					
mRNA	0.90*	0.91*	0.86*	0.91**	0.85*
protein	0.84*	0.97**	0.88*	0.91*	0.99**
mTOR					
mRNA	-0.95**	-0.92**	-0.91*	-	-
protein	-0.91*	-0.85*	-0.83*	-	-
**PI3K-RNAi**					
PI3K					
mRNA	0.97**	0.97**	0.92*	0.99**	0.96**
protein	0.91*	0.94**	0.99**	0.96**	0.97**
mTOR					
mRNA	-0.91*	-0.88*	-0.97**	-	-
protein	-0.93**	-0.94**	-0.98**	-	-
**mTOR-RNAi**					
PI3K					
mRNA	0.96**	0.83*	0.93**	0.92**	0.93**
protein	0.91*	0.99**	0.92**	0.86*	0.92**
mTOR					
mRNA	-0.92*	-0.94**	-0.92*	-	-
protein	-0.95**	-0.83*	-0.96*	-	-

*P<0.05, **P<0.01 (Pearson's linear correlation analysis).

**Table 4:** Analysis of the correlation between mRNA and protein expression levels of PI3K, mTOR, RhoA, p-RhoA protein, and mean fluorescence intensity (MFI) and percent phagocytosis (r value).

Index/Group	PI3K		mTOR		RhoA		p-RhoA protein
	mRNA	Protein	mRNA	Protein	mRNA	Protein	
MFI							
Blank control	0.92*	0.93**	- 0.83*	- 0.91*	0.95**	0.84*	0.86*
Negative control	0.93**	0.92**	- 0.92*	- 0.91*	0.89*	0.98**	0.91*
PI3K-RNAi	0.90*	0.84*	- 0.85*	- 0.87*	0.85*	0.92*	0.88*
mTOR-RNAi	0.93**	0.85*	- 0.83*	- 0.91*	0.92**	0.98**	0.90*
Phagocytosis (%)							
Blank	0.95**	0.93**	-0.89*	-0.97**	0.95**	0.96**	0.97**
Negative control	0.94**	0.82*	-0.96**	-0.94**	0.99**	0.90*	0.97**
PI3K-RNAi	0.99**	0.90*	-0.88*	-0.92*	0.96**	0.97**	0.90*
mTOR-RNAi	0.99**	0.82*	-0.87*	-0.98**	0.94**	0.92**	0.90**

*P<0.05, **P<0.01 (Pearson's linear correlation analysis).

## Discussion

The surface receptors on AM recognize pathogens and bind to ligands to activate relevant signaling pathways, such as PI3K and RhoA, which modulate cytoskeletal rearrangements to engulf exogenous particles (8). In this study, it was shown that the PI3K-mTOR-RhoA signaling pathway inhibited cytoskeletal rearrangement by affecting macrophage phagocytosis. After recognition by pathogen recognition receptors (PRRs), PI3K-Akt initiates a series of phosphorylation cascades and activates mTOR, which subsequently affects cytoskeletal rearrangements by regulating the activity of RhoA; whereby mTORC2 promotes the activation of RhoA and enhances the cytoskeletal rearrangements; whereas mTORC1 reduces the activity of RhoA and inhibits the cytoskeletal rearrangements (14–16).

In this study, the expressions of PI3K and mTOR were silenced by RNAi to explore their specific roles in PI3K/mTOR/RhoA signaling pathway, in order to study the effects of this pathway on the phagocytic capacity of macrophages. RNAi involves the introduction of exogenous double-stranded RNA into cells to induce the degradation of its corresponding homologous mRNA, thereby post-transcriptionally silencing its target gene (17). RNAi has rapidly become a standard biological procedure as siRNAs yield greater efficiencies than conventional antisense oligonucleotides and ribozymes, and RNAi-mediated knockdown of target genes is relatively feasible compared to gene knockouts via homologous recombination (18). In this study, we successfully silenced the target genes by transfecting shRNA recombinant lentiviral vectors into cells with gene-silencing efficiencies >80%. The attempt made by Jacinto et al. (19) to silence the expression of mTOR using siRNA led to the upregulation of RhoA. Gan et al. (20) found that Type I PI3K could directly activate mTORC2, and the inhibition of Type I PI3K using LY294002 led to the downregulation of mTORC2 and RhoA. In this study, we found that the expressions of mTOR, RhoA, and p-RhoA were downregulated following the silencing of PI3K, possibly because Type I PI3K, whose expression was silenced in this study, can directly activate mTORC2. Both PI3K and mTOR are located upstream to RhoA, and thus may exert varying effects on RhoA. It has also been found that PI3K could directly activate RhoA (19,21). Moreover, Jeruschke et al. (22) found that the inhibition of mTOR using the immunosuppressant everolimus could partially restore the activity of RhoA, which is consistent with this study that the expressions of RhoA and p-RhoA were upregulated after silencing the expression of mTOR.

Our correlation analysis showed that PI3K was positively correlated with RhoA, whereas mTOR was negatively correlated with RhoA. PI3K positively regulates RhoA and is positively correlated with the phagocytic capacity of macrophages, whereas mTOR negatively regulates RhoA and is negatively correlated with the phagocytic capacity of macrophages. Therefore, the downregulation of RhoA and RhoA phosphorylation after silencing the expression of PI3K might be attributed to the fact that the positive induction of PI3K on RhoA is greater than the negative effect of mTOR on RhoA, which is consistent with the findings reported in previous studies (19,23), but is different from others (24). The reason could be that mTOR exists in two complexes referred to as mTOR complex 1 and 2 (mTORC1 and mTORC2, respectively), which play different roles in different signaling pathway (25). mTORC1 phosphorylates proteins such as eukaryotic initiation factor 4E binding protein 1 (4E-BP1), the 70-kDa ribosomal protein S6 kinase 1 (p70S6K1), and uncoordinated-51-like kinase 1 (ULK1), whereas mTORC2 phosphorylates proteins such as Akt (26,27). The repressive effect of RhoA·GTP occurs through a decrease in the amount of Rheb (Ras-homolog enriched in brain) · GTP available to stimulate mTORC1 through RhoA · GTP-mediated repression of mTORC2 leading to reduced Akt signaling to TSC2 (28).

Cytoskeleton comprises actin, microfilaments, and intermediate filaments. Actin plays important roles in cell division, movement, and phagocytosis (29). Phagocytosis refers to the process in which after the recognition of exogenous particles by surface receptors on macrophages, the aggregation of actin at the plasma membrane leads to the formation of pseudopod projections that wrap the phagocytosed particle, followed by the closure of phagocytic cups, depolymerization of actin, and formation of phagolysosomes (30). RhoA is a key protein that regulates cytoskeletons, whereby it can activate its downstream effectors Rho-associated protein kinase (ROCK) and mammalian diaphanous (mDia) to promote myosin contraction and actin rearrangement, which are required for the formation of stress fibers (31). During the complement receptor 3 (CR3)-mediated phagocytosis, RhoA promotes the polymerization of actin and microtubules to form phagocytic cups that engulf and digest the phagocytosed particle (32). Salinas et al. reported that the actin polymerization in HeLa cells was obstructed exhibiting reduced phagocytic capacity against rickettsia after silencing the RhoA gene. Inhibition of PI3K led to blocking of AKT phosphorylation and attenuated the downregulation of F-actin genes in pulmonary arterial smooth muscle cell (PASMC) (33). Schlam et al. (34) found that the process of phagocytosis in macrophages was interrupted after inhibiting PI3K, whereby the pseudopod projections were blocked by the formation of shallow phagocytic cups and continuous aggregation of actin, which could not be depolymerized. In this study, we showed that following the silencing of PI3K gene, the macrophages displayed a reduced phagocytic capacity with disordered cytoskeleton arrangements, short pseudopod projections, and a reduced number of stress fibers. The cells exhibited a more pronounced cellular deformation after silencing the mTOR gene with an increased number of stress fibers and enhanced phagocytic capacity. The results showed that the inhibiting of PI3K could inactivate the Akt-RhoA GTPase-actin rearrangement cascade, but the inhibiting of mTOR could upregulate phosphorylation of RhoA.

In summary, both PI3K and mTOR genes were successfully silenced in this study via lentiviral transfection method. We showed that PI3K/mTOR/RhoA signaling pathway affected the phagocytic capacity of macrophages by modulating the cytoskeletal changes. PI3K enhanced the phagocytic capacity of macrophages by positively regulating phosphorylation levels of RhoA and promoting a proper cytoskeletal rearrangement. On the other hand, mTOR attenuated the phagocytic capacity of macrophages by negatively regulating phosphorylation levels of RhoA and blocking a proper cytoskeletal rearrangement. Therefore, this study highlighted the possibility of developing a specific inhibitor against mTOR to improve the phagocytic capacity of macrophages and reduce the frequency of acute exacerbations in patients with COPD. Moreover, the complex and heterogenic enzymatic pathway of mTOR and the use of mTOR inhibitors for COPD therapy need a more in-depth knowledge of mTOR signaling (35).

In this study, we only silenced the expressions of PI3K and mTOR by RNAi, but did not silence RhoA. The mechanism of the interaction between RhoA and mTOR is not clear. Because we did not determine Akt and F-actin, the mechanism of AM and cytoskeletal rearrangement for PI3K/mTOR/RhoA pathway is unclear after the expressions of PI3K and mTOR were silenced, and their effect on interaction of mTORC1 and mTORC2 with RhoA is unclear in COPD. Therefore, further research is needed, and should include animal models.
